# Surgical Timing and Approach in Gartland Type III Supracondylar Humerus Fractures in Children: Does After-Hours Surgery Influence Clinical and Radiological Outcomes? A Retrospective Cohort Study

**DOI:** 10.3390/jcm15103673

**Published:** 2026-05-10

**Authors:** Erkan Servet, Murat Düzgün, Musa Alperen Bilgin, Cagrı Karabulut, Beytullah Unat, Nevzat Gönder

**Affiliations:** 1 Department of Orthopaedics and Traumatology, Faculty of Medicine, Gaziantep University, Gaziantep 27310, Turkey; dr.erkanservet@yahoo.com (E.S.); muratduzgun1995@gmail.com (M.D.); 2 Department of Orthopaedics and Traumatology, T.C. Ministry of Health Aksaray Training and Research Hospital, Aksaray 68000, Turkey; alpperren@gmail.com; 3 Department of Orthopaedics and Traumatology, T.C. Ministry of Health Pazarcık State Hospital, Kahramanmaraş 46700, Turkey; drcagrikarabulut@hotmail.com; 4 Department of Orthopaedics and Traumatology, School of Medicine, Gaziantep Islam Science and Technology University, Gaziantep 27000, Turkey; unatbeytullah@gmail.com

**Keywords:** supracondylar humerus fracture, Gartland Type III, after-hours surgery, open reduction, working hours

## Abstract

**Background/Objectives**: Gartland Type III supracondylar humerus fractures (SCHFs) represent the most surgically challenging pediatric elbow injuries, yet controversy persists regarding whether the timing of surgery, specifically after-hours versus working-hours operations, influences clinical and radiological outcomes. This study aimed to compare the functional, cosmetic, and radiological outcomes of Gartland Type III SCHFs managed during working hours versus after hours, with a secondary analysis incorporating the surgical approach (open vs. closed reduction). **Methods**: A retrospective cohort study was conducted, including 91 pediatric patients who underwent surgical treatment for Gartland Type III SCHFs between January 2020 and June 2025. Patients were stratified into working-hours (*n* = 48) and after-hours (*n* = 43) groups. Outcomes were assessed using Flynn’s criteria, radiological parameters, range-of-motion measurements, and complication rates. A secondary subgroup analysis was performed across four groups formed by combining surgical timing and approach. **Results**: The mean patient age was 70.36 ± 32.97 months with a mean follow-up of 28.07 ± 14.66 months. The time to surgery was significantly shorter in the working-hours group (median 16.0 h; IQR 13.5–20.0) compared with the after-hours group (median 20.0 h; IQR 17.0–27.0) (*p* = 0.009). No significant differences were observed between the two groups with respect to functional outcomes, cosmetic outcomes, radiological parameters, or overall complication rates (all *p* > 0.05). However, four-group subgroup analysis revealed a significant difference in Flynn’s functional outcomes (*p* = 0.019), with the after-hours open reduction subgroup demonstrating a lower rate of excellent results (76.92%) compared with the remaining subgroups (96.88–100%). **Conclusions**: Working-hours versus after-hours surgical timing alone does not significantly alter clinical or radiological outcomes in Gartland Type III SCHFs. However, the combination of after-hours surgery with open reduction appears to be associated with inferior functional outcomes and a trend toward higher complication rates, suggesting that open reduction for complex fractures should preferably be performed during working hours when optimal theatre conditions and experienced senior surgical teams are readily available.

## 1. Introduction

Supracondylar humerus fractures (SCHFs) are the most common surgically treated fractures in the pediatric population, accounting for more than 60% of all elbow fractures and approximately 16–20% of all pediatric fractures [1,2]. These injuries are frequent in children between 3 and 10 years of age and typically result from a fall on an outstretched hand in the extension mechanism [1]. The complex anatomy of the distal humerus, the proximity of critical neurovascular structures, and the fracture’s propensity for malunion, particularly in the form of cubitus varus, render Gartland Type III injuries among the most technically demanding conditions in pediatric orthopedic surgery [3,4].

The Gartland classification, initially proposed in 1959 and subsequently refined, remains the most widely adopted system for characterizing extension-type SCHFs [5]. Type III fractures are defined by complete cortical disruption of both the anterior and posterior cortices, accompanied by tearing of the anterior periosteum. The posterior periosteal hinge typically remains intact. This residual posterior periosteal sleeve is biomechanically important, as it serves as a soft-tissue tether that the surgeon may exploit during reduction by maximally flexing the elbow, thereby tensioning the intact posterior envelope and facilitating coronal and sagittal alignment prior to pin insertion [6]. This complete displacement necessitates surgical intervention under general anesthesia, most commonly employing closed reduction and percutaneous K-wire fixation (CRPP) or, when closed reduction fails, open reduction with percutaneous pinning (ORPP) [6,7,8,9].

Subsequently, Leitch et al. introduced Type IV as a distinct subtype, characterized by multidirectional instability resulting from complete circumferential disruption of the periosteal sleeve, with the distal fragment being displaceable in both flexion and extension. These injuries are notoriously unstable and frequently require open reduction owing to their tendency to redisplace intraoperatively despite adequate fluoroscopic alignment [9].

A perennial debate in pediatric orthopedic practice concerns the optimal timing of surgical intervention. Emergency departments frequently receive children with these injuries during evenings, nights, weekends, and holidays, periods conventionally defined as ‘after hours’—generating pressure to operate immediately or defer to the next available elective list. Proponents of immediate intervention argue that progressive soft-tissue swelling, vascular compromise, and increasing fracture instability over time may complicate the reduction and compromise the overall outcome [10]. Conversely, advocates for selective deferral emphasize that non-urgent cases can be safely managed the following morning with the benefit of fully staffed theatres, experienced senior surgeons, and optimal anesthetic support [11,12].

The contemporary evidence base on this question, while substantial, remains heterogeneous and largely inconclusive. A systematic review by Terpstra et al. identified only six comparative studies in the published literature, none of which demonstrated statistically significant differences in functional outcomes between office-hours and after-hours surgery [13]. Nevertheless, individual studies have reported higher rates of malunion in late-night subgroups; increased risk of paresthesia during overnight procedures; and superior fixation quality when surgery is performed during the day [13,14,15]. A further dimension of complexity is introduced by the type of reduction required: open reduction inherently carries greater technical demands and a higher complication profile, and its interaction with surgical timing has not been systematically examined [16,17].

In a large international survey of 184 surgeons from the European Paediatric Orthopaedic Society (EPOS), surgical timing was identified as one of the principal ongoing controversies in the management of SCHFs, alongside pin configuration and operative approach [7]. Against this backdrop, real-world institutional data from adequately powered cohorts are needed to provide surgeons with evidence-based guidance for decision-making at the time of patient admission.

The primary objective of this study was to compare the clinical and radiological outcomes of Gartland Type III SCHFs managed during working hours versus after hours in a pediatric orthopedic surgery setting. A secondary objective was to determine whether the combined effect of surgical timing and reduction technique (open vs. closed) further stratified the outcome differences.

## 2. Materials and Methods

### 2.1. Study Design and Ethics

This was a single-centre retrospective cohort study conducted at the Department of Orthopedics and Traumatology. This study was performed in line with the principles of the Declaration of Helsinki. Approval was granted by the Gaziantep University Non-Interventional Research Ethics Committee (Date: 18 February 2026-No. 2026/53).

### 2.2. Patient Selection

The medical records of all patients who underwent surgical treatment for SCHFs at our institution between January 2020 and June 2025 were reviewed. Inclusion criteria were as follows: (1) age < 18 years; (2) confirmed Gartland Type III extension-type SCHF; (3) treatment with either CRPP or ORPP; and (4) a minimum of 6 months of postoperative follow-up. Exclusion criteria were as follows: (1) Gartland Type I or Type II fractures; (2) flexion-type SCHFs; (3) associated neurovascular injury requiring emergent vascular exploration; (4) pathological fractures; (5) Type IV fractures; and (6) incomplete medical records or loss to follow-up. Although Type IV fractures were excluded from the present cohort, their recognition is critical, as their management may differ substantially from that of extension Type III injuries.

Patients with absolute vascular compromise (a cold, pale, pulseless hand with delayed capillary refill, or any clinical or Doppler evidence of brachial artery injury) were excluded from analysis, as such cases mandate emergent surgical intervention regardless of administrative timing categories. The ‘pink, pulseless’ hand, in which collateral circulation maintains adequate distal perfusion despite an absent radial pulse, poses a particular diagnostic challenge: misinterpretation of capillary refill in this scenario can lead to inappropriate surgical deferral with potentially catastrophic consequences, including ischemic contracture or limb loss. For this reason, every Type III SCHF in our institution undergoes meticulous serial neurovascular assessment, including radial pulse palpation, capillary refill, and monitoring of hand temperature and oxygen saturation. A low threshold for Doppler ultrasound or surgical exploration is used when concern persists.

### 2.3. Grouping Strategy

Eligible patients were divided into two primary groups based on the institutional definitions of surgical shift periods: (1) working-hours group—operations initiated between 08:00 and 17:59 on weekdays; and (2) after-hours group—operations initiated outside these hours, including evenings (18:00–23:59), nights (00:00–07:59), weekends, and public holidays. Working hours were defined according to our institutional protocol as 08:00–17:59 on weekdays, during which fully staffed elective and trauma operating lists are operational with senior pediatric orthopedic and anesthetic team coverage. After 18:00, the institution transitions to a reduced on-call staffing model, which is operationally defined here as ‘after hours’. For the secondary analysis, four subgroups were formed by cross-tabulating surgical timing with surgical approach: (a) working hours + CRPP; (b) working hours + ORPP; (c) after hours + CRPP; and (d) after hours + ORPP.

K-wire fixation was performed using either 1.6 mm or 2.0 mm Kirschner wires, with the diameter selected based on patient age and the size of the distal humeral fragment. Specifically, 1.6 mm wires were used predominantly in children younger than six years, and 2.0 mm wires were used in older or larger children.

### 2.4. Outcome Measures

The primary outcome was the functional and cosmetic result, as assessed by Flynn’s criteria, which evaluates loss of elbow flexion and carrying-angle deviation from the contralateral side. Outcomes were classified as excellent (0–5°), good (6–10°), fair (11–15°), or poor (>15°) for each parameter [18]. Secondary outcomes included the following: (1) radiological parameters—Baumann angle, humeroulnar angle, and humerotrochlear angle—measured on standardized anteroposterior and lateral elbow radiographs; (2) range of motion (ROM) of the affected elbow (flexion, extension, supination, and pronation) compared with the contralateral side; (3) overall and individual complication rates; and (4) time to surgery from injury.

Additional variables recorded included patient age, sex, affected side, mechanism of injury, fracture classification (open/closed), associated injuries, K-wire configuration, K-wire removal time, and duration of splinting.

### 2.5. Sample Size Calculation

Sample size was calculated using G*Power (version 3.9.1; Franz Faul, Universität Kiel, Kiel, Germany). Based on the published literature reporting an effect size of d = 0.62 for between-group differences in time to surgery, a two-tailed independent-samples comparison with a type I error rate (α) of 0.05 and statistical power (1 − β) of 0.80 required a minimum of 42 patients per group (total *n* = 84).

### 2.6. Statistical Analysis

All statistical analyses were performed using SPSS Statistics, version 22.0 (IBM Corporation, Armonk, NY, USA). Continuous variables are presented as the mean ± standard deviation (SD) or median with interquartile range (IQR), as appropriate. Normality was assessed using the Shapiro–Wilk test. Between-group comparisons of continuous variables were performed using the Mann–Whitney U test (two groups) or the Kruskal–Wallis H test (four subgroups). Categorical variables are expressed as frequencies and percentages, and between-group associations were analyzed using the Fisher–Freeman–Halton exact test. A two-tailed *p*-value < 0.05 was considered statistically significant.

## 3. Results

### 3.1. Patient Demographics and Baseline Characteristics

A total of 91 patients met all inclusion criteria and were enrolled in the study. The mean age at the time of injury was 70.36 ± 32.97 months (median: 66 months; range: 19–152 months). The cohort comprised 58 male (63.74%) and 33 female (36.26%) patients. The left elbow was involved in 57 cases (62.64%), and the right elbow was injured in 33 cases (36.26%); one patient (1.10%) sustained bilateral fractures.

The most prevalent mechanism of injury was a simple ground-level fall (46 patients; 50.55%), followed by falls from furniture (16; 17.58%), a bicycle (13; 14.29%), in the playground (7; 7.69%), from a height (4; 4.40%), and down a staircase (4; 4.40%) and a motor vehicle collision (1; 1.10%). The vast majority of fractures were closed (87; 95.60%); four patients sustained open injuries (Gustilo Type I: 3; Gustilo Type II: 1). Associated ipsilateral upper-limb fractures were present in four patients (2.20% forearm both-bone fractures; 2.20% distal radius fractures). Detailed baseline data are presented in Table 1.

### 3.2. Surgical Data and Timing

Surgery was performed during working hours in 48 patients (52.75%) and after hours in 43 patients (47.25%). The overall mean time from injury to surgical intervention was 23.12 ± 21.93 h. Time to surgery was significantly shorter in the working-hours group (median 16.0 h; IQR 13.5–20.0 h) compared with the after-hours group (median 20.0 h; IQR 17.0–27.0 h) (Mann–Whitney U test, *p* = 0.009).

The principal causes of surgical delay in this cohort were inter-hospital transfer from peripheral centres (41% of patients), late presentation to the emergency department, theatre availability outside working hours, and pre-operative optimization (e.g., fasting status or anesthetic clearance). No patient experienced a delay due to the deliberate selection of intervention after hours over a working-hours list when the clinical condition permitted earlier intervention.

CRPP was the primary surgical technique in 62 patients (68.13%), ORPP was required in 28 patients (30.77%), and a combined approach was used in 1 patient (1.10%). Conversion to open reduction was indicated in 28 patients (30.77%) for the following reasons: failure of closed reduction after multiple fluoroscopically guided attempts (*n* = 18), soft-tissue interposition (*n* = 8), and complex rotational displacement or late presentation with established swelling (*n* = 2). Some patients had overlapping indications. The relatively higher open reduction rate compared with some published series may reflect our institutional role as a tertiary referral centre for complex and delayed-presentation Type III injuries, as well as the exclusion of Type I and II fractures from the cohort denominator. The most frequently utilized K-wire configuration was two lateral pins plus one medial pin (62 patients; 68.13%), followed by one lateral plus one medial pin (15; 16.48%), two lateral plus two medial pins (8; 8.79%), and other configurations (6; 6.60%). Mean K-wire removal time was 6.02 ± 1.14 weeks, and mean splinting duration was 4.23 ± 0.75 weeks.

### 3.3. Primary Outcome: Working-Hours vs. After-Hours Comparison

Comparison between the working-hours and after-hours groups revealed no statistically significant differences with respect to age, follow-up duration, K-wire removal time, splinting duration, carrying angle, ROM (flexion, extension, supination, and pronation), or radiological parameters (Baumann angle, humeroulnar angle, and humerotrochlear angle) (all *p* > 0.05). Similarly, no significant intergroup differences were detected for sex, laterality, presence of associated injuries, injury mechanism, fracture type (open/closed), surgical technique, K-wire count, Flynn’s cosmetic score, Flynn’s functional score, or overall complication rate (all *p* > 0.05). The overall Flynn cosmetic score was excellent in 90 patients (98.90%) and good in 1 patient (1.10%). Flynn’s functional outcomes were excellent in 87 patients (95.60%), good in 3 patients (3.30%), and poor in 1 patient (1.10%). Aggregate surgical data and outcome parameters are summarized in Table 2.

### 3.4. Secondary Outcome: Four-Subgroup Analysis

Four subgroups were formed by cross-tabulating surgical timing and reduction techniques: working hours + CRPP (WH + CRPP; *n* = 32; 35.16%), working hours + ORPP (WH + ORPP; *n* = 16; 17.58%), after hours + CRPP (AH + CRPP; *n* = 30; 32.97%), and after hours + ORPP (AH + ORPP; *n* = 13; 14.29%). No significant intergroup differences were identified for any continuous variable (all *p* > 0.05).

However, a Kruskal–Wallis comparison of Flynn’s functional outcomes across the four subgroups revealed a statistically significant difference (*p* = 0.019). The rate of excellent functional outcomes was 96.88% in the WH + CRPP group, 100% in both the WH + ORPP and AH + CRPP groups, and 76.92% in the AH + ORPP group. In the AH + ORPP subgroup, 15.38% of patients achieved a good outcome, and 7.69% (one patient) achieved a poor outcome.

Regarding complications, 77 patients (84.62%) experienced no postoperative complications. The overall complication profile included elbow ROM restriction (4; 4.40%), loss of reduction requiring revision surgery (2; 2.20%), spontaneous K-wire migration (2; 2.20%), K-wire subcutaneous penetration requiring a second surgical procedure (2; 2.20%), transient postoperative ulnar neuropraxia resolving at follow-up (1; 1.10%), intraoperative K-wire breakage (1; 1.10%), cubitus varus with reduction loss (1; 1.10%), and fracture-side hyperextension (1; 1.10%). The overall complication rate did not differ significantly between the working-hours and after-hours groups (*p* = 1.000). In the four-subgroup analysis, AH + ORPP exhibited a numerically higher complication rate (5/13; 38.46%) compared with the remaining subgroups (WH + CRPP: 9.38%; WH + ORPP: 0%; AH + CRPP: 6.67%); however, this difference did not achieve statistical significance (*p* = 0.068). Subgroup outcome data are displayed in Table 3.

## 4. Discussion

The central finding of this study is that surgical timing in the form of working hours versus after hours does not independently affect the clinical or radiological outcomes of surgically treated Gartland Type III SCHFs. This finding is consistent with the majority of recent evidence in the literature [11,12,19,20]. What our four-subgroup analysis adds to this conclusion is an important nuance: when after-hours surgery is combined with the requirement for open reduction, outcomes are meaningfully inferior, with a significantly lower rate of excellent functional results and a trend toward a higher complication rate. This combined interaction has rarely been evaluated systematically and constitutes the primary novel contribution of the present study.

The absence of a difference in outcomes between working-hours and after-hours CRPP is reassuring and agrees with the findings of Mehlman et al., who reported no difference in perioperative complications based on surgical timing in their landmark JBJS study. Similarly, Bales et al. found comparable postoperative carrying angles, ROM, and complication profiles irrespective of time-to-surgery [19,21]. The meta-analysis by Farrow et al., encompassing 1735 SCHFs, also demonstrated that delayed intervention did not negatively affect outcomes in the absence of vascular compromise, with a mean time to surgery of 91.8 h in the delayed group [20]. Our own time-to-surgery figure of 23.12 h overall is within the generally accepted safe window.

The findings regarding the interaction between after-hours timing and open reduction are more clinically significant. Open reduction inherently introduces a greater technical challenge, requiring precise identification of neurovascular structures, meticulous handling of soft tissues, and adequate visualization. These are all factors that may be compromised in fatigued theatre teams operating under suboptimal late-night conditions. Albrahim et al. similarly reported that the majority of documented postoperative complications in their retrospective series occurred in cases performed after daytime working hours and those requiring open reduction [22]. Paci et al. found that while overall outcomes did not differ by shift, late-night operations (23:00–05:59) were associated with higher malunion rates: an observation potentially reflecting the additive effect of surgeon fatigue and increasing technical complexity [14]. Taken together, these findings support a stratified approach to surgical timing in Gartland Type III SCHFs: closed reduction in the absence of a neurovascular compromise may safely be deferred to the next available working-hours list, whereas cases anticipated to require open reduction should preferentially be performed during working hours, when senior pediatric orthopedic surgical expertise, fully staffed theatre teams, and optimal anesthetic support are readily available. To mitigate surgeon and team fatigue, the relative complexity of open reduction, as well as the availability of experienced personnel, should each be explicitly weighed in the decision to proceed with procedures after hours, particularly when open reduction is anticipated.

Although our overall median time-to-surgery of 16 h in the working-hours group is well below the mean of 91.8 h reported in the delayed groups of the Farrow et al. meta-analysis, it is admittedly longer than the <12-h threshold proposed by Mehlman et al. as a potential quality benchmark. It is broadly consistent with the <18-h benchmark used in some institutional quality indicators [19,20]. The relatively prolonged interval in our cohort largely reflects the regional referral pattern of our tertiary centre, which serves as a major referral hub for southeastern Anatolia and frequently receives patients transferred from peripheral hospitals after preliminary radiographic and clinical evaluation. Importantly, however, multiple subsequent studies, including those by Bales et al., Sullivan et al., and Okkaoglu et al., have failed to demonstrate a clinically meaningful difference in outcomes based on the 12-h cutoff in the absence of vascular compromise [11,12,21]. Our findings are therefore consistent with the contemporary view that the question of relevant timing does not reflect absolute hours from injury but rather the careful selection of cases that can safely be deferred to optimal operating conditions.

The literature on surgical approach is relevant here. Anterior, posterior, medial, and lateral approaches to open reduction each carry distinct complication profiles. A study comparing medial and lateral surgical approaches in Type III SCHFs found no difference in functional outcomes. Instead, it found that the medial approach offered advantages in terms of a lower risk of iatrogenic ulnar nerve injury and superior cosmesis of the incision [23]. A meta-analysis of CRPP versus ORIF encompassing 581 patients found no significant difference in Flynn’s functional or cosmetic outcomes between the two modalities. However, this analysis did not stratify patients by the timing of the open procedure, precluding firm conclusions about the interaction between timing and approach [8].

Neurovascular complications deserve specific attention in the context of surgical timing. Iatrogenic ulnar nerve injury remains the most commonly reported neurological complication associated with medial pin placement. There have been reported incidence rates of up to 5.2% [24]. In our series, transient ulnar neuropraxia was observed in one patient (1.10%) and resolved completely at follow-up, consistent with the history of this complication described by Eidelman et al. [25]. Compartment syndrome, despite its rarity with an estimated incidence of 0.1–0.3%, represents the most feared acute complication and is recognized as an absolute indication for emergent surgical intervention irrespective of timing [26]. We deliberately excluded patients with vascular compromise from our analysis to isolate the timing effect in the elective-urgent scenario that is most relevant to everyday clinical decision-making.

The K-wire configuration debate is separately relevant to outcomes. Our predominant configuration, comprising two lateral pins plus one medial pin (68.13%), achieves a balance between the biomechanical superiority of crossed-pin constructs, which demonstrate higher resistance to rotational and varus forces, and the risk of iatrogenic ulnar nerve injury with medial pin insertion [27,28]. A contemporary meta-analysis of randomized controlled trials by Carrazzone et al. concluded that crossed fixation achieves lower rates of reduction loss and provides superior stability, albeit at the cost of a higher risk of nerve injury; this trade-off must be individualized for each patient [29]. The recent finding that inserting the medial pin first under an identified tactile nerve reduces intraoperative reduction loss may further refine practice patterns in experienced centres [30]. The biomechanical principle of dual-column stabilization is particularly critical in completely displaced Type III injuries, in which lateral-only pinning may be associated with insufficient bone engagement in one of the two fragments and consequent loss of reduction. Our institutional preference for crossed (medial–lateral) pin configurations in this fracture subset, reflected in the 68.13% rate of two-lateral + one-medial fixation, was driven by this rationale.

Radiological assessment in our cohort included the Baumann, humeroulnar, and humerotrochlear angles—three well-validated indices of coronal and sagittal reduction quality [31,32]. No significant differences were identified between the two primary groups for any of these parameters. This suggests that satisfactory reduction can be achieved regardless of shift timing when the indication for surgery is correctly identified and the appropriate technique is applied.

The present study has several limitations that should be acknowledged. First, its retrospective design introduces inherent selection bias, and the absence of randomization precludes causal inference. Secondly, the sample size of 91 patients, while exceeding our pre-specified calculation of statistical power for the two-group comparison, results in limited power within the four subgroups. This underpowered analysis likely explains why the AH + ORPP complication rate of 38.46% did not achieve formal statistical significance (*p* = 0.068) despite its clinical relevance. Third, surgeon experience was not formally quantified or controlled for, and the level of senior oversight during after-hours operations may have varied across the study period. In observational studies, this factor has been shown to influence outcomes independently [22]. Fourth, we acknowledge that the 18:00 threshold is somewhat later than the 16:00–17:00 cutoff adopted in some other international centres, and this institutional variability is a methodological consideration that may affect direct comparability with other published works. Finally, the relatively short minimum follow-up of 6 months may not capture the development of late deformities such as cubitus varus, which may manifest over multiple years as growth proceeds. Despite these limitations, the present study is strengthened by the exclusive focus on Gartland Type III injuries, the highest-acuity subset of SCHFs, and the novel four-subgroup analytical framework.

## 5. Conclusions

In children with Gartland Type III supracondylar humerus fractures managed without neurovascular compromise, surgical timing in the form of working-hours versus after-hours treatment does not independently determine clinical, cosmetic, or radiological outcomes. These findings support the safety of selective deferral to the following working-hours list for appropriate patients when immediate theatre resources, experienced surgical personnel, or optimal anesthetic support are not readily available.

However, the combination of after-hours surgery with open reduction was associated with a significantly lower rate of excellent functional outcomes and a clinically relevant but statistically nonsignificant increase in complications. This interaction suggests that complex Gartland Type III cases anticipated to require open reduction should be prioritized for working-hour operating lists whenever the clinical condition permits. When after-hours open reduction is unavoidable, every effort should be made to ensure the availability of a senior, experienced surgeon and a fully equipped operative team. Future prospective multicenter studies incorporating objective measures of surgeon fatigue and team composition are needed to establish definitive guidelines.

## Figures and Tables

**Table 1:** Patient demographics and baseline characteristics (*n* = 91).

Characteristic	Value (*n* = 91)
Age (months), mean ± SD	70.36 ± 32.97
Median (range)	66 (19–152)
Follow-up (months), mean ± SD	28.07 ± 14.66
Median (range)	26 (6–69)
Sex, *n* (%)	
Male	58 (63.74%)
Female	33 (36.26%)
Fracture Side, *n* (%)	
Left	57 (62.64%)
Right	33 (36.26%)
Bilateral	1 (1.10%)
Injury Mechanism, *n* (%)	
Simple fall	46 (50.55%)
Fall from couch/furniture	16 (17.58%)
Fall from bicycle	13 (14.29%)
Fall in playground	7 (7.69%)
Fall from height	4 (4.40%)
Fall from stairs	4 (4.40%)
Motor vehicle accident	1 (1.10%)
Fracture Type, *n* (%)	
Closed fracture	87 (95.60%)
Open fracture (Type 1)	3 (3.30%)
Open fracture (Type 2)	1 (1.10%)
Associated Injuries, *n* (%)	
None	87 (95.60%)
Forearm both-bone fracture	2 (2.20%)
Distal radius fracture	2 (2.20%)

SD: standard deviation.

**Table 2:** Surgical data and overall clinical outcomes (*n* = 91).

Surgical Parameter	Value (*n* = 91)
Surgical Timing, *n* (%)	
Working hours	48 (52.75%)
After hours	43 (47.25%)
Time to Surgery (hours), *n* (%)	
Overall, mean ± SD	23.12 ± 21.93
Working-hours group, median (IQR)	16.0 (13.5–20.0)
After-hours group, median (IQR)	20.0 (17.0–27.0)
*p*-value (Mann–Whitney U)	0.009
Surgical Technique, *n* (%)	
Closed reduction + K-wire fixation	62 (68.13%)
Open reduction + K-wire fixation	28 (30.77%)
Combined (closed + open)	1 (1.10%)
K-wire Configuration, *n* (%)	
2 Lateral + 1 Medial	62 (68.13%)
1 Lateral + 1 Medial	15 (16.48%)
2 Lateral + 2 Medial	8 (8.79%)
Other configuration	6 (6.60%)
Postoperative Follow-up Parameters	
K-wire removal time (weeks), mean ± SD	6.02 ± 1.14
Splint duration (weeks), mean ± SD	4.23 ± 0.75
Flynn’s Cosmetic Outcomes, *n* (%)	
Excellent	90 (98.90%)
Good	1 (1.10%)
Flynn’s Functional Outcomes, *n* (%)	
Excellent	87 (95.60%)
Good	3 (3.30%)
Poor	1 (1.10%)
Complications, *n* (%)	
No complication	77 (84.62%)
Elbow range-of-motion restriction	4 (4.40%)
Loss of reduction → revision surgery	2 (2.20%)
Spontaneous K-wire migration	2 (2.20%)
K-wire subcutaneous migration → 2nd surgery	2 (2.20%)
Postoperative ulnar neuropraxia (resolved)	1 (1.10%)
Intraoperative K-wire breakage	1 (1.10%)
Cubitus varus + reduction loss	1 (1.10%)
Fracture-side hyperextension	1 (1.10%)

Mann–Whitney U test; IQR: interquartile range; SD: standard deviation; CRPP: closed reduction and percutaneous pinning; ORPP: open reduction and percutaneous pinning.

**Table 3:** Four-subgroup analysis of Flynn’s functional outcomes and complication rates.

Outcome	WH + CRPP (*n* = 32)	WH + ORPP (*n* = 16)	AH + CRPP (*n* = 30)	AH + ORPP (*n* = 13)
Flynn’s Functional				
Excellent, *n* (%)	31 (96.88%)	16 (100%)	30 (100%)	10 (76.92%)
Good, *n* (%)	1 (3.13%)	0 (0%)	0 (0%)	2 (15.38%)
Poor, *n* (%)	0 (0%)	0 (0%)	0 (0%)	1 (7.69%)
*p*-value *	*p* = 0.019 (Kruskal–Wallis)			
Complications				
Any complication, *n* (%)	3 (9.38%)	0 (0%)	2 (6.67%)	5 (38.46%)
*p*-value *	*p* = 0.068 (Fisher–Freeman–Halton)			

WH: working hours; AH: after hours; CRPP: closed reduction and percutaneous pinning; ORPP: open reduction and percutaneous pinning. * Kruskal–Wallis test (functional outcomes); Fisher–Freeman–Halton exact test (complications).

## Data Availability

Data are available on request due to restrictions (e.g., privacy, legal, or ethical reasons).

## Abbreviations

The following abbreviations are used in this manuscript:

SCHFs	Gartland Type III supracondylar humerus fractures
CRPP	Percutaneous K-wire fixation
ORPP	Open reduction with percutaneous pinning
WH	Working hours
AH	After hours
